# Sex Differences in Energetic Cost of Walking and Later-Life Function and Physical Activity Patterns

**DOI:** 10.1093/geroni/igaa057.615

**Published:** 2020-12-16

**Authors:** Matthew Peterson, Kira Byrd, Andrew Frohle

**Affiliations:** 1 Campbell University, Buies Creek, North Carolina, United States; 2 Wright State University, Dayton, Ohio, United States

## Abstract

Aerobic fitness is a strong predictor of functional independence with aging. Variability in aerobic fitness is due, in part, to the efficiency of the movement of body mass while ambulating [energetic cost of walking (ECoW)]. ECoW may serve as a low burden measure of fitness compared to volitional treadmill tests. We assessed the predictive ability of ECoW with later-life function and physical activity participation in healthy adults. N=75 (age = 54.7 +/- 9.2; 52% female) completed a treadmill test with indirect calorimetry, during which time a warm-up of 1.5 mph at 0% grade was uniform. ECoW was quantified as steady state volume of oxygen consumption (ml/kg/min) subtracted by the calculated metabolic cost of walking at given speed and grade (=7.5 ml/kg/min). Later-life (median=9 years follow-up) function was assessed using the SF-36 physical functioning subscale, and the Baecke questionnaire for physical activity. Mean EcoW was similar to calculated cost (difference = -0.14 ml/kg/min) but had large variation (SD = 2.7; range = -4.4 to +17 ml/kg/min). In females, higher ECoW was predictive of better later-life function (b=1.9; p=0.04) and higher physical activity levels (b=0.09; p=0.001). In males, lower ECoW was predictive of higher physical activity levels only (b=-0.04; p=0.01). All models controlled for % body fat, age, and comorbidities. Surprisingly, in females, higher ECoW was associated with better later-life health outcomes. We hypothesize that this phenomenon may be similar to the obesity paradox, in that relatively higher non-metabolic tissue in females may serve as a stimulus for muscle and functional preservation.

